# When Uncontrolled Diabetes Mellitus and Severe COVID-19 Converge: The Perfect Storm for Mucormycosis

**DOI:** 10.3390/jof7040298

**Published:** 2021-04-15

**Authors:** Teny M. John, Ceena N. Jacob, Dimitrios P. Kontoyiannis

**Affiliations:** 1 Department of Infectious Diseases, Infection Control and Employee Health, The University of Texas MD Anderson Cancer Center, Houston, TX 77030, USA; TMJohn1@mdanderson.org; 2 Department of Internal Medicine, The University of Texas Medical Center in Houston, Houston, TX 77093, USA; Ceena.N.Jacob@uth.tmc.edu

**Keywords:** COVID-19, diabetes mellitus, mucormycosis

## Abstract

Mucormycosis (MCR) has been increasingly described in patients with coronavirus disease 2019 (COVID-19) but the epidemiological factors, presentation, diagnostic certainty, and outcome of such patients are not well described. We review the published COVID-19-associated mucormycosis (CAMCR) cases (total 41) to identify risk factors, clinical features, and outcomes. CAMCR was typically seen in patients with diabetes mellitus (DM) (94%) especially the ones with poorly controlled DM (67%) and severe or critical COVID-19 (95%). Its presentation was typical of MCR seen in diabetic patients (mostly rhino-orbital and rhino-orbital-cerebral presentation). In sharp contrast to reported COVID-associated aspergillosis (CAPA) cases, nearly all CAMCR infections were proven (93%). Treating physicians should have a high suspicion for CAMCR in patients with uncontrolled diabetes mellitus and severe COVID-19 presenting with rhino-orbital or rhino-cerebral syndromes. CAMR is the convergence of two storms, one of DM and the other of COVID-19.

## 1. Introduction

Coronavirus disease 2019 (COVID-19) pandemic, caused by severe acute respiratory syndrome virus 2 (SARS-CoV-2), has affected more than 130 million people worldwide, accounting for over 2.8 million deaths on the day of this report. Although aspergillosis has been reported to complicate severe COVID-19 (the entity is coined COVID-19-associated aspergillosis or CAPA), the pathophysiology and the true incidence of CAPA remains debatable, as very few CAPA cases are biopsy-documented [1,2].

In addition, Mucorales infections are emerging as a matter of concern in COVID-19, as poorly controlled diabetes mellitus (DM) and other co-morbidities are risk factors for both severe COVID-19 and mucormycosis (MCR) [3] and the use of corticosteroids to treat severe/critical COVID-19 is a well-known risk factor for MCR [4]. To that end, we reviewed the cases of MCR in patients with COVID-19, discuss the pathophysiology of MCR in the setting of COVID-19 and contrast the firmness of COVID-19-associated mucormycosis (CAMCR) to the ambiguities of CAPA diagnosis.

## 2. Materials and Methods

We searched PubMed (1 December 2019–9 April 2021) using the search terms “COVID-19”, “SARS CoV-2”, “novel coronavirus infection”, and “mucormycosis”, ”Mucorales”, “non-aspergillus mold”, “*Mucor*”, “*Rhizopus*”, “*Rhizomucor*”, “*Cunninghamella*”, and “*Lichtehimia*” (“*Absidia*”). We included only cases or case series reports containing adequate information about risk factors, clinical presentation, course, diagnosis, treatment, and outcome. Proven MCR was defined as per the EORTC-MSG criteria [5]. Since “probable” invasive fungal disease (IFD) in EORTC/MSG criteria applies only to immunocompromised hosts, we adapted the proposed definitions for CAPA to further define “probable” and “putative” MCR cases [6]. Patients who had a syndrome consistent with MCR such as rhino-orbital disease, with a positive culture from the nasal cavity were considered to have “probable” MCR. Patients with COVID-19 and cavitary pneumonia with the isolation of Mucorales from respiratory secretions were considered to have “putative” MCR. Mucormycosis was considered “concurrent” if evidence of infection was present on admission and “sequential” if a diagnosis was made 72 h after COVID-19 diagnosis.

## 3. Results

We identified 43 such patients; 41 cases with documented MCR (proven in 38, probable in 3, Table 1) and 2 putative cases (see Supplementary Table S1 and references). We identified five patients with rhino-orbital disease, and one patient with rhino-orbital-cerebral disease, which was not included as there was no culture or histopathology documentation, given the fact that COVID-19 rarely can mimic rhino-orbital disease due to ophthalmic vein thrombosis [7]. Thirty-four (83%) were males with a median age of 55 years (IQR 46–61). More than two-thirds of the patients (29/41, 71%) were reported from India. Although 40 (40/41, 98%) patients had risk factors for severe COVID-19, only two patients had EORTC risk factors for IFD. Of the 35 patients with information on glycemic status, 33 (33/35, 94%) had DM, with a mean HbA1C of 10 (in patients who had an HbA1c value available), and 22 (22/33, 67%) had uncontrolled DM before admission; three (3/33, 9%) patients had new-onset DM. Of the eighteen patients with DM and report of blood glucose and acid status, 8 (8/18, 44%) were in diabetic ketoacidosis (DKA) at the time of presentation. Among the 22 patients with COVID-19 severity information, thirteen (13/22, 59%) patients had ‘critical’ COVID-19, requiring mechanical ventilation/non-invasive ventilation, while eight had ‘severe’ COVID-19 and one patient had ‘mild’ COVID-19. In the 9 patients with available renal function data or creatinine value, 7 (78%) hadrenal failure on admission, including one patient who was on dialysis at the time of admission. Although neutrophil and lymphocyte counts were available in only 5 and 4 patients respectively, only one had neutropenia (neutrophil count < 500/microliter), and 3 had lymphocytopenia (total lymphocyte count < 1000/microliter). When Mucorales were identified to the genus level (13 patients), *Rhizopus* species were isolated in 10 (77%).

Sixteen patients (16/41, 39%) had ”concurrent” MCR, while the remaining patients (25/41) were diagnosed with MCR after a mean of 22 days (SD of 24). All but one patient with sequential MCR received systemic corticosteroids, while three of these patients received tocilizumab. Thirty-one of 41 patients (76%) had either rhino-orbital-cerebral (11, 27%), rhino-cerebral (3, 7%), or rhino-orbital disease (17, 41%), 3 (7%) had sinusitis alone, 3 (7%) had pulmonary MCR, and one each had cutaneous, palatal, gastrointestinal and disseminated MCR. Importantly, all 33 patients with DM had sinusitis, palatal, rhino-orbital, or rhino-orbital-cerebral disease. Co-infections were uncommon; 2 patients had a bacterial sinus infection (one with *Pseudomonas* and one with S*taphylococcus aureus*), and two had a sinus culture growing *Aspergillus*. MCR diagnosis was made antemortem in 39/41 (95%) and by autopsy in 2. For patients with antemortem diagnosis, MCR was established by biopsy in 31, and tissue culture in 18. In two patients who underwent postmortem, sequencing (2/2) and PCR (1/1) aided in the diagnosis. Of the 39 patients with antemortem MCR, amphotericin B-based treatment was administered in 38 (with posaconazole in 7 and with isavuconazole in 1). Of the 15 patients with information about the timing of anti-fungal therapy in relation to diagnosis of MCR, six received empiric treatment and nine following the diagnosis of MCR. Thirty-three (80%) of these patients underwent adjunct surgery (sinus and thoracic cavity debridement, orbital exenteration, decortication, and lung resection). In-hospital mortality was 49% (20/41), with an average time of death 19 days post-MCR diagnosis (information available only in 7 patients).

## 4. Discussion

Although our review is subject to publication biases, several observations emerged. First, MCR in patients with COVID-19 is a well-documented disease (41/47 of reported cases (87%) had documented MCR), in sharp contrast with the experience with CAPA. Specifically, the incidence of proven, or probable CAPA according to the recent ECMM/ISHAM consensus criteria [6] was only 6.1% in cohort CAPA studies that had a denominator [8]. 

Second, the majority of cases (71%) are reported from India. This finding is not surprising as India has the highest burden of MCR in the world with an estimated prevalence of 140 cases per million population [9]. Additionally, India has the second-largest number of adults aged 20–79 years with DM [10]. In fact, DM is the single most common risk factor for mucormycosis in India, being reported in over 50% of cases of MCR. In a recent nationwide multi-center study on MCR in India, 57% of patients had uncontrolled diabetes mellitus and 18% had diabetic ketoacidosis [11]. The number of uncontrolled DM we observed in our review was similar to this number (67%) although there were more patients with DKA (44%). 

Third, the clinical presentation of MCR in our review was quite consistent with the picture of MCR in DM where rhino-orbital or rhino-cerebral disease predominates [12]. DM, a “classic” risk factor for MCR, is associated with increased morbidity and mortality in COVID-19 [13]. As with any other serious infection, patients with COVID-19 are predisposed to DKA. Evidence suggests SARS CoV-1 induces damage of pancreatic islets resulting in acute diabetes and DKA [14]. This is a possible explanation for the “diabetogenic state” in SARS CoV-2 infection, as there is a high expression of angiotensin-converting enzyme 2 receptors in pancreatic islets, along with increased insulin resistance due to cytokine storm [15]. Prevalence of DM (31%) and DKA (2%) in COVID-19 were higher compared to the national prevalence of type 2 DM and DKA in the general population in a UK study (7%) [16]. Recently, euglycemic DKA is also being reported in COVID-19 patients [17]. The frequent use of corticosteroids that exacerbated glucose homeostasis, may have predisposed patients to MCR. Corticosteroid use is a key risk factor for opportunistic mycoses, including MCR [4].

In addition to hyperglycemia, an alteration of iron metabolism occurs in severe COVID-19 [18]. Severe COVID-19 is a hyper-ferritinemic syndrome, but whether high ferritin is a marker of a severe systemic disease versus a modulator of pathophysiology is not known. Irrespective of its role, high ferritin levels lead to excess intracellular iron that generates reactive oxygen species resulting in tissue damage. Cytokines, especially IL-6, due to severe infection and DKA, stimulate ferritin synthesis and downregulate iron export resulting in intracellular iron overload, further exacerbating the process [18]. The resultant tissue damage leads to the release of free iron into the circulation [19]. Iron overload and excess free iron seen in acidemic states are one of the key and unique risk factors for MCR [20].

Another possible explanation for the association between COVID-19 and MCR is the “endothelialitis” observed in severe COVID-19. Autopsy series have shown more severe pulmonary vascular endothelial injury and new vessel growth in patients who died of COVID-19 than patients who died of influenza A (H1N1) [21]. Another postmortem series noted widespread endothelial injury in patients who died of multi-organ failure [22]. Endothelial adhesion and penetration are critical early steps in MCR [20]. Interestingly, acidemic states and hyperglycemia induce the enthodelial receptor glucose-regulated protein (GRP 78) and the Mucorales adhesin spore coat protein homologs (CotH), creating a “perfect storm” for increased adhesion and penetration of Mucorales to the endothelium. Of interest, GRP 78 has been postulated as one of the receptors responsible for SARS-CoV-2 entry [23].

We acknowledge the limitations of our review, which is the lack of a denominator, making it impossible to calculate MCR incidence in patients with COVID-19. It is possible that reported MCR cases may be an underrepresentation of the actual problem due to difficulty making a histopathological or microbiological diagnosis in patients with a contagious infection in a pandemic setting. Another limitation is the inability to assess attributable mortality of MCR due to the lack of appropriate controls. Lastly, the nature of our study does not allow us to determine if uncontrolled diabetes plus COVID-19 is associated with a higher risk of MCR than diabetes alone.

## 5. Conclusions

MCR appears to be the intersection of two crises: the one of COVID-19 and the other of poorly controlled DM in the setting of the pandemic. In sharp contrast to CAPA, most CAMCR cases are well documented. Clinicians should have a high index of suspicion and keep CAMCR in the differential of a severely ill patient with COVID-19 and DM, especially if rhino-orbital or rhino-cerebral presentations are noted. Craniofacial pain without swelling can be a neuropathic pain in DM patients with COVID-19 and by itself without facial asymmetry or necrosis should not be suggestive of MCR in these patients [24]. Although the impact of preemptive antifungal therapy remains unproven, hyperglycemia control seems important for prevention and management in MCR [12].

## Figures and Tables

**Table 1:** Characteristics of the 41 patients with COVID-19 associated mucormycosis (CAMCR).

		Documented Mucormycosis (*n* = 41, 95%) ^i^
Sex–Male, *n* (%)		34 (83)
Age (in years) median (IQR)		55 (46–61)
Presence of risk factors for severe COVID-19, *n*(%)		40 (98)
EORTC risk factors, *n* (%)		2 (5)
Diabetes mellitus, *n* (%) ^ii^		33 (94)
HbA1C (mean, SD) ^iii^		10.5 (4.4)
Presence of DKA on admission, *n* (%) ^iv^		8 (44)
Renal failure, *n* (%) ^v^		5 (83)
COVID-19 severity ^vi^	Mild, *n* (%)	1 (5)
	Severe, *n* (%)	13 (59)
	Critical, *n* (%)	8 (36)
ICU admission ^vii^		15 (68)
Systemic corticosteroids, *n* (%)		36 (88)
Timing of mucormycosis diagnosis		
Concurrent, n (%)		16 (39)
Sequential, *n* (%)		25 (61)
Clinical syndrome, *n* (%)	Rhino-orbital-cerebral	11 (27)
	Rhino-orbital	17 (41)
	Rhino-cerebral	3 (7)
	Sinusitis alone	3 (7)
	Pneumonia	3 (7)
	Other ^viii^	4 (10)
Microbiological diagnosis, *n* (%)	Stain	40 ^ix^ (98)
	Tissue PCR/Sequencing	2 (5)
	Tissue culture	18(44)
Histopathological diagnosis, *n* (%) ^x^		31 (76)
Genera, *n* (%)	*Rhizopus* spp.	10 (24)
	*Lichteimia* spp.	1 (2)
	*Mucor* spp	2 (5)
	Mucorales, unspecified	28 (68)
Co-infection, *n* (%) ^xi^		3 (7)
Mucorales-active, anti-fungal treatment, *n* (%) ^xii^		38 (93)
In-hospital mortality, *n* (%)		20 (49)
Day of death after MCR diagnosis (mean, SD) ^xiii^		19, 15

Abbreviations: EORTC, European Organization for Research and Treatment of Cancer; COVID-19, Coronavirus disease 2019; HbA1C, hemoglobin A1C DKA, diabetic ketoacidosis; ICU, intensive care unit; PCR, polymerase chain reaction. **^i^** Total cases reported, 43; Documented CAMCR, 41-includes “Proven” in 38 and “Probable” in 3; 39 cases diagnosed ante mortem and 2 cases diagnosed post mortem; ^ii^ Information available only in 35 patients; ^iii^ Information available in only 3/9 patients with diabetes mellitus; ^iv^ Information on blood glucose levels and acid status available only in 18 patients; ^v^ Renal function data or creatinine available only in 6 patients; ^vi^ Severity reported only in 22 patients; ^vii^ At the time of CAMCR diagnosis, information available in only 22 patients; ^viii^ Includes one patient each with gastric, cutaneous, palatal and disseminated MCR; ^ix^ Information not available in 1 patient; ^x^ autopsy findings were; Patient 11: vasculocentric and disseminated pattern involving lungs, hilar lymph nodes, brain, heart and kidneys; lungs showed severe marked pulmonary edema and hemorrhage with focal frank necrosis, a mixture of exudative and organizing focal diffuse alveolar damage (DAD), kidneys showed acute kidney injury and myoglobin casts, heart showed fibrinous pericarditis, and brain showed hemorrhagic infarction. There was evidence of multi-organ thrombo-emboli. Patient 12: necrotic lung with fungal hyphae, no signs of dissemination; ^xi^ One patient each had *Pseudomonas*, and *Staphylococcus aureus* and 2 patients had *Aspergillus* sinus infection; ^xii^ Included amphotericin B (AM-B) (in 10) with posaconazole (in 6), 3 patients did not receive AMB; one died immediately after diagnostic procedure and 2 diagnoses were post-mortem; ^xiii^ not reported in thirteen patients.

## Supplementary Materials

The following are available online at https://www.mdpi.com/article/10.3390/jof7040298/s1, Table S1: Detailed clinical characteristics of forty-one patients with documented COVID-19 associated mucormycosis; proven (*n* = 38) and probable (*n* = 3) and 2 patients with putative mucormycosis
