# Supplementary material for: When Uncontrolled Diabetes Mellitus and Severe COVID-19 Converge: The Perfect Storm for Mucormycosis
Source: J Fungi (Basel). 2021 Apr 15;7(4):298. doi: 10.3390/jof7040298 (PMC8071133; doi:10.3390/jof7040298)
Supplement: Supplementary file 1 [file jof-07-00298-s001.zip › jof-1159820-supplementary.pdf]

	Patient 1	Patient 2	Patient 3	Patient 4-8	Patient 9	Patient 10	Patient 11	Patient 12	Patient 13	Patients 14-30	Patients 31-36	Patient 37	Patient 38	Patient 39	Patient 40	Patient 41	Patient 42	Patient 43
**Author / year**	Placik etal. 2020 (1)	Werthman-Ehrenreich. 2020 (2)	Monte Junior etal. 2020 (3)	Sen etal. 2021 (4)	Mekonnen etal.2020 (5)	Mehta etal. 2020 (6)	Hanley etal. 2020 (7)	Zurl etal. 2021 (8)	Waizel-Haiat etal. 2021 (9)	Moorthy etal. 2021 (10)	Sarkar etal. 2021 (11)	Kanwar etal. 2021 (12)	Pauli etal. 2021 (13)	Dallalzadeh etal.2021 (14)	Karimi-Galougahi et al.2021 (15)	Khatri A. etal. 2021 (16)	Pasero etal. 2020 (17)	Garg etal., 2021 (18)
**Country of reporting**	USA	USA	Brazil	India	USA	India	UK	Austria	Mexico	India	India	USA	Brazil	USA	Iran	USA	Italy	India
**Sex**	Male	Female	Male	Male (n=5)	Male	Male	Male	Male	Male	Male 15, Female 2	Male 4, Female 2	Male	Female	Male	Female	Male	Male	Male
**Age (in years)**	49	33	86	58 (mean)	60	60	22	53	24	54.8	44	56	50	48	61	68	66	55
**Risk factors for severe Covid-19**[i]	None	DM, HTN, Asthma	HTN	DM (n=5), HTN (n=1), CAD (n=1)	DM, Asthma, HTN	DM	Severe obesity (BMI 48.8)	MDS, AML	Obesity	DM (n=15)	DM (n=6)	ESRD	DM	DM	DM	Heart transplant	HTN (on ACE)	DM, HTN, CAD, cardiomyopathy, ESRD
**EORTC risk factors**	None	None	None	None	None	None	None	Yes, neutropenia	None	None	NA	None	None	None	NA	Yes	None	None
**Occupational exposure**	NA	NA	NA	NA	NA	NA	NA	NA	NA	NA	NA	NA	NA	NA	NA	NA	NA	NA
**Diabetes mellitus**	No	Yes, new onset	No	Yes (n=5), uncontrolled (n=3)	Yes, uncontrolled	Yes	NA	NA	Yes, new onset	Yes, uncontrolled (n=15)	Yes (n=6)	NA	Yes, uncontrolled	Yes	Yes, New Onset	Yes	No	Yes, uncontrolled
**HbA1C**	NA	NA	NA	8.8 (mean) (n=3)	14	NA	NA	NA	NA	NA	NA	NA	NA	NA	NA	NA	NA	5.3 (low due to anemia)
**DKA on admission**	NA	Yes	NA	Yes (n=2)	Yes	Yes	NA	NA	Yes	NA	Yes (n=2)	NA	No	No	NA	No	No	No
**Initial X-ray/CT**	Bilateral patchy opacities	Left lower lobe consolidation	Ground glass opacities with consolidation	Not available	ARDS	Extensive peripheral ground glass and consolidative opacities	Bilateral air space consolidation	Bilateral peripheral ground glass opacities	Peripheral ground-glass and consolidative opacities	NA	NA	NA	NA	ARDS	NA	central and peripheral ground glass opacities	NA	Bilateral diffuse infiltrates
**X-ray/ CT (at the time of mucormycosis)**	Pneumothorax, bullae/cavity concerning for BPF, necrotic empyema	NA	NA	Right cavernous sinus and fronto parietal lobe involvement (n=1)	NA	NA	Hemorrhagic Right MCA infarct	Increase in bilateral infiltrates, pleural effusion	Striation of extra-conal fat in the face and peri-orbital region	NA	Sinusitis ± orbital ± intracranial involvement	Bilateral air space density and pleural effusion	No significant alteration in the maxilla	MRI with Right sino-nasal cavity and anterior skull base extending to bilateral Frontal lobes	Complete Opacification of Right Maxillary Sinus and Anterior Ethmoidal air cells with internal foci of air that was representative of acute infarct	6.4 x 5.3 cm fluid collection in anterior chest wall at previous IABP catheter site with subclavian artery pseudo-aneurysm	Dense consolidation, buried cavity, pleural effusion	Right upper zone cavity
**Sinus involvement**	NA	Yes	NA	Yes (n=5), and Sino-cutaneous fistula (n=1)	Yes, pan sinusitis	Yes, pan sinusitis	NA	NA	Yes (maxillary, ethmoid and sphenoid)	Yes (n=17)	Yes (n=6)	NA	NA	Right sinonasal cavity	Yes(Maxillary and Ethmoidal)	No	Yes	NA
**Covid severity**[ii]	Severe (5 L O2, involvement of > 50% lung)	Severe (ICU)	Critical (mechanical ventilation) (ICU)	Severe (n=4), NA =1	Critical (mechanical ventilation) ICU	Severe (ICU)	Critical (mechanical ventilation, vaso pressors) (ICU)	Critical (NIV) ICU)	Critical (ICU)	NA	Critical (n=6)	Severe	Mild	Critical (ICU)	NA	Critical	Critical (mechanical ventilation)	Severe
**Covid-19 specific therapy**	Remdesivir	Remdesivir, CCP	Oseltamivir, Azithromycin	NA (n=5)	CCP		NA	NA	NA	NA	Remdesivir (n=2)	CCP	No	CCP	Remdesivir, Interferon alpha	CCP	HCQS, Lopinavir-ritonavir	Remdesivir
**Systemic steroids, duration (if available)**	Dexamethasone	No	Hydrocortisone	Methylprednisolone/Prednisone/Dexamethasone (n=5)	Dexamethasone	Methylprednisolone/ Dexamethasone	NA	Prednisolone (high dose)	NA	Yes (n=17)	Steroids (n=6)	Methyl prednisone	No	Dexamethasone	Yes	Methylprednisolone/ Prednisone (for gout)	No	Dexamethasone x 14 days
**Other therapies**	Tocilizumab	No	No	NA	No	Tocilizumab	NA	Tocilizumab	NA	NA	NA	Tocilizumab	No	NA	NA	No	No	No
**Renal failure**	NA	Yes	Yes	NA	No	Yes	Yes	NA	Yes	NA	NA	Yes	No	NA	NA	Yes	Yes	Yes
**Renal replacement therapy**	None	No	No	NA	No	No	NA		NA	NA	NA	Yes (present on admission)	No	NA	NA	No	Yes	Yes (present on admission)
**Vasopressors**	None	No	Yes	NA	No	No	Yes	Yes	NA	NA	NA	NA	No	NA	NA	No	Yes	No
**Time from Covid-19 to mucormycosis (in days)**	14	Concurrent	Concurrent	Concurrent (n=1), Sequential (n=4)	7	10	Concurrent	Concurrent	Concurrent	Concurrent (n=4)	Concurrent (n=6)	16	8	6	21	90	17	17
**Clinical syndrome**	Necrotizing pneumonia with bronchopleural fistula	Rhino-orbital-cerebral disease	Gastric mucor	Rhino-orbital-cerebral	Rhino-orbital	Rhino-orbital	Disseminated (lungs, hilar lymph nodes, heart - fibrinous pericarditis, brain and kidneys)	Fungal pneumonia with effusion	Rhino-orbital	Sinusitis alone (n=3), Rhino-orbital (n=6), Rhino-orbital-cerebral (n=5), Rhino-cerebral (n=3)	Rhino-orbital (n=5), Rhino-orbital-cerebral (n=1)	Necrotizing pneumonia with empyema	Palatal ulcer	Rhino-orbital	Rhino- orbital	Cutaneous	Cavitary pneumonia with pleural effusion	Cavitary pneumonia with pleural effusion
**Clinical specimen**	Necrotic lung	Sinus tissue	Gastric biopsy	Orbital (n=4) and sinus tissue (n=5)	Sinus tissue	Sinus tissue	Full autopsy	Full autopsy	Sinus sample	Sinus tissue	Sinus tissue	Lung tissue	Palate tissue	Pus culture	Sinus tissue	Tissue debridement	BAL	Sputum culture

**Fungal markers**	NA	NA	NA	NA	NA	NA	NA	Serum beta-d-glucan 178 pg/mL, Serum Aspergillus GM antigen negative	NA	NA	NA	NA	NA	NA	NA	Serum beta-d-glucan 43 pg/mL, Serum *Aspergillus* GM antigen index < 0.5	NA	Serum beta-d-glucan 189 pg/mL, Serum *Aspergillus* GM antigen 0.18
**Neutropenia**	NA	Absent (22,000)	Absent (11,200)	NA	NA	NA	Absent	Yes (ANC < 500)	Absent	NA	NA	Absent	NA	NA	NA	Absent	Absent (10,300)	Absent
**Lymphopenia (TLC<1000/microL)**	NA	1370	580	NA	NA	Mild (value not reported)	NA	100	NA	NA	NA	Yes (582)	NA	NA	NA	NA	NA	NA
**Microbiological diagnosis**																		
**- stain**	Yes	Yes	Yes	Yes (n=5)	Yes	Yes	Yes	Yes	Yes	Yes	Yes	Yes	Yes	Yes	NA	Yes	Yes	Yes
**- PCR**	NA	NA	NA	NA	NA	NA	Yes	NA	NA	NA	NA	NA	NA	NA	NA	NA	NA	Not done
**- sequencing**	NA	NA	NA	NA	NA	NA	Yes	Yes	NA	NA	NA	NA	NA	NA	NA	NA	NA	Not done
**- culture**	Yes	Yes	NA	No	Yes	Yes	Not reported	NA	Yes	NA	Yes	Yes	No	Yes	NA	Yes	Yes (rapidly growing cotton-candy colony)	Yes
**Histopathological diagnosis**	Yes	NA	Yes	Yes	Yes	Yes	Yes	Yes	No	Yes	NA	Yes	Yes	No	Yes	Yes	Negative	No
**Genera**	Rhizopus sp.	Mucorale, unspecified	Mucorale, unspecified	Mucorale, unspecified (n=5)	Rhizopus	Mucorale, unspecified	Mucorale, unspecified	Rhizopus microsporus	Lichteimia (Absidia) spp.	Mucorale, unspecified	Rhizopus (n=4), Mucor (n=2)	Rhizopus azygosporus	Mucorale, unspecified	Rhizopus spp.	Mucorale , Unspecified	Rhizopus microsporus	Rhizopus sp.	Rhizopus microsporus
**Disease classification**[iii]	Documented, Proven	Documented, Proven	Documented, Proven	Documented, Proven (n=5)	Documented, Proven	Documented, Proven	Documented, Proven (post-mortem)	Documented, Proven (post mortem)	Documented, Probable	Documented, Proven (n=17)	Documented, Proven (n=4), Probable (n=2)	Documented, Proven	Documented, Proven	Documented, Proven	Documented, Proven	Documented, Proven	Putative	Putative
**Co-infection (including CMV reactivation)**	Not reported	Staphylococcus aureus	None	Pseudomonas (n=1), Aspergillus (n=1)	None	Not available	Not reported	NA	No	Aspergillus (n=1)	NA	No	No	No	No	No	Not reported	No
**Co-colonization**	No		None	None	None	None	Not reported	NA	*K.pneumoniae, E.cloacae, E. fecalis*	NA	NA	No	No	No	No	No	Candida glabrata on siuns sample	No
**Anti-fungal therapy**	AM-B	AM-B	None	Liposomal AM-B + voriconazole/ Posaconazole (n=5)	Liposomal AM-B+ caspofungin /Posaconazole	Amphotericin B	None	None	Amphotericin B	Amphotericin B	Amphotericin B	Amphotericin B	Amphotericin B	AMB/Isuvaconazole	Systemic antifungals (Unspecified)	AM-B + Posaconazole	AM-B/ Isavuconazole	Amphotericin B
**Adjunct surgery**	Resection	Sinus debridement	None	Exenteration (n=2), sinus debridement (n=3)	Sinus debridement	None	NA	NA	None	FESS (n=17), Maxillectomy (n=11), Exenteration (n=11)	Maxillectomy (n=3), FESS (n=1)	Robotic decortication	Debridement	None	Endonasal Endoscopic Debridement + Right eye exenteration	Thoracic cavity debridement	None	None
**Outcome**	Died (day 21)	Died (day 26)	Died (7 days)	Survived (n=5)	Died (day 31)	Died (day 16)	Died (day 27)	Died (day 24)	Died	Died (n=6)	Died (n=3)	Died (day 33)	Survived	Died	Survived	Died	Died (day 62)	Survived
**Time of death after MCR diagnosis (days)**	5	26	2		29	6	Not applicable	Not applicable	NA	NA	NA	17		NA	NA	NA	45	

Abbreviations: NA, not available; EORTC, European Organization for Research and Treatment of Cancer; DM, diabetes mellitus; DKA, diabetic ketoacidosis; HTN, hypertension; CAD, coronary artery disease; BMI, body mass index; MDS, myelodysplastic syndrome; AML, acute myeloid leukemia; MCR, mucormycosis; HbA1C, hemoglobin A1C; CT, computerized tomography; ARDS, acute respiratory distress syndrome; NIV, non-invasive ventilation; BPF, broncho-pleural fistula; MCA, middle cerebral artery; ICU, intensive care unit; CCP, COVID-19 convalescent plasma; AM-B, amphotericin B.

[i] Risk factors for severe Covid-19 include cardiovascular disease including coronary artery disease (CAD), diabetes mellitus (DM), hypertension (HTN), chronic lung disease including asthma, chronic kidney disease, cancer, obesity and smoking

[ii] Covid severity - Mild: no pneumonia or mild pneumonia (lung infiltrates < 50%), Severe: dyspnea, respiratory rate ≥ 30 / min, blood oxygen saturation ≤ 93 %, PaO2 / FiO2 < 300 and/ or lung infiltrates > 50 % within 24-48 hours, Critical: respiratory failure, septic shock and/or multi-organ dysfunction

[iii] Proven - if histopathologic, cytopathologic or direct microscopic examination of a specimen obtained by obtained by needle aspiration or biopsy in which hyphae or melanized yeast-like forms are seen accompanied by evidence of associated tissue damage OR Recovery of a hyaline or pigmented mold by culture of a specimen obtained by a sterile procedure from a normally sterile and clinically or radiologically abnormal site consistent with an infectious disease process, excluding BAL fluid, a paranasal or mastoid sinus cavity specimen, and urine OR Blood culture that yields a mold (eg, Fusarium species) in the context of a
compatible infectious disease process OR Amplification of fungal DNA by PCR combined with DNA sequencing when molds are seen in formalin-fixed paraffin-embedded tissue, Putative - if none of the crteria are met but Mucor is attributed as a pathogen and patient was treated for it.

1. Placik DA, Taylor WL, Wnuk NM. Bronchopleural fistula development in the setting of novel therapies for acute respiratory distress syndrome in SARS-CoV-2 pneumonia. Radiol Case Rep. 2020;15(11):2378-81.
2. Werthman-Ehrenreich A. Mucormycosis with orbital compartment syndrome in a patient with COVID-19. Am J Emerg Med. 2020.
3. Monte Junior ESD, Santos M, Ribeiro IB, Luz GO, Baba ER, Hirsch BS, et al. Rare and Fatal Gastrointestinal Mucormycosis (Zygomycosis) in a COVID-19 Patient: A Case Report. Clin Endosc. 2020;53(6):746-9.
4. Sen M, Lahane S, Lahane TP, Parekh R, Honavar SG. Mucor in a Viral Land: A Tale of Two Pathogens. Indian J Ophthalmol. 2021;69(2):244-52.
5. Mekonnen ZK, Ashraf DC, Jankowski T, Grob SR, Vagefi MR, Kersten RC, et al. Acute Invasive Rhino-Orbital Mucormycosis in a Patient With COVID-19-Associated Acute Respiratory Distress Syndrome. Ophthalmic Plast Reconstr Surg. 2020.
6. Mehta S, Pandey A. Rhino-Orbital Mucormycosis Associated With COVID-19. Cureus. 2020;12(9):e10726.
7. Hanley B, Naresh KN, Roufosse C, Nicholson AG, Weir J, Cooke GS, et al. Histopathological findings and viral tropism in UK patients with severe fatal COVID-19: a post-mortem study. The Lancet Microbe. 2020;1(6):e245-e53.
8. Zurl C, Hoenigl M, Schulz E, Hatzl S, Gorkiewicz G, Krause R, et al. Autopsy Proven Pulmonary Mucormycosis Due to Rhizopus microsporus in a Critically Ill COVID-19 Patient with Underlying Hematological Malignancy. J Fungi (Basel). 2021;7(2).
9. Waizel-Haiat S, Guerrero-Paz JA, Sanchez-Hurtado L, Calleja-Alarcon S, Romero-Gutierrez L. A Case of Fatal Rhino-Orbital Mucormycosis Associated With New Onset Diabetic Ketoacidosis and COVID-19. Cureus. 2021;13(2):e13163.
10. Moorthy A, Gaikwad R, Krishna S, Hegde R, Tripathi KK, Kale PG, et al. SARS-CoV-2, Uncontrolled Diabetes and Corticosteroids-An Unholy Trinity in Invasive Fungal Infections of the Maxillofacial Region? A Retrospective, Multi-centric Analysis. J Maxillofac Oral Surg. 2021:1-8.
11. Sarkar S, Gokhale T, Choudhury SS, Deb AK. COVID-19 and orbital mucormycosis. Indian J Ophthalmol. 2021;69(4):1002-4.
12. Kanwar A, Jordan A, Olewiler S, Wehberg K, Cortes M, Jackson BR. A Fatal Case of Rhizopus azygosporus Pneumonia Following COVID-19. J Fungi (Basel). 2021;7(3).
13. Pauli MA, Pereira LdM, Monteiro ML, de Camargo AR, Rabelo GD. Painful palatal lesion in a COVID-19 patient. Oral Surgery, Oral Medicine, Oral Pathology and Oral Radiology. 2021.
14. Dallalzadeh LO, Ozzello DJ, Liu CY, Kikkawa DO, Korn BS. Secondary infection with rhino-orbital cerebral mucormycosis associated with COVID-19. Orbit. 2021:1-4.
15. Karimi-Galougahi M, Arastou S, Haseli S. Fulminant mucormycosis complicating coronavirus disease 2019 (COVID-19). Int Forum Allergy Rhinol. 2021.
16. Khatri A, Chang K-M, Berlinrut I, Wallach F. Mucormycosis after Coronavirus disease 2019 infection in a heart transplant recipient – case report and review of literature. Journal of Medical Mycology. 2021.
17. Pasero D, Sanna S, Liperi C, Piredda D, Branca GP, Casadio L, et al. A challenging complication following SARS-CoV-2 infection: a case of pulmonary mucormycosis. Infection. 2020.

18. Garg D, Muthu V, Sehgal IS, Ramachandran R, Kaur H, Bhalla A, et al. Coronavirus Disease (Covid-19) Associated Mucormycosis (CAM): Case Report and Systematic Review of Literature. Mycopathologia. 2021(Feb 5):1-10.
